# The relationship between neuroticism as a personality trait and mindfulness skills: a scoping review

**DOI:** 10.3389/fpsyg.2024.1401969

**Published:** 2024-11-15

**Authors:** Natalia Angarita-Osorio, Rosa M. Escorihuela, Toni Cañete

**Affiliations:** ^1^Department of Psychiatry and Forensic Medicine, Faculty of Medicine, Universitat Autònoma de Barcelona, Bellaterra, Spain; ^2^Mental Health Research Group, Hospital del Mar Research Institute, Barcelona, Spain; ^3^Centre for Biomedical Research in Mental Health Network (CIBERSAM), Madrid, Spain; ^4^Institut de Neurociències, Universitat Autònoma de Barcelona, Bellaterra, Spain

**Keywords:** mindfulness, neuroticism, scoping review, personality traits, mindfulness-based interventions

## Abstract

**Introduction:**

In recent decades, researchers have assessed the relationship between mindfulness and personality traits, including neuroticism, a known target in mental health associated with the development of mental health disorders and physical illnesses. The main aim of mindfulness practice is to help individuals develop the ability to regulate and accept their experiences, emotions, and thoughts. Therefore, it could be suggested that mindfulness may be useful in reducing the expression and negative experience of neuroticism. The aim of our review was to assess the relationship between neuroticism and mindfulness.

**Methods:**

We conducted a scoping review of the literature in December 2023, using the databases PubMed and PsycINFO.

**Results:**

Forty-nine studies were included in the review, with four common themes identified: (i) mental health, (ii) cognitive outcomes, (iii) physiological symptoms, and (iv) mindfulness-based interventions. Across most of the studies, mindfulness negatively correlated with neuroticism, supporting the idea that mindfulness may be useful in reducing neuroticism and its negative effects on mental and physical health.

**Discussion:**

While several limitations were identified, the overall results are promising. Future research in this area should focus on overcoming the current limitations to provide a better understanding of the relationship between mindfulness and neuroticism.

## 1 Introduction

In recent decades, there has been a growing interest and body of literature regarding the impact and relationship of mindfulness with several psychological constructs. Among those, personality traits described as patterns of thoughts, feelings, and behaviors that tend to be stable throughout our lifetime (VandenBos, 2007). These traits have been combined and organized into several models that propose different structures for personality.

Eysenck (1947) proposed a personality trait theory based on two primary dimensions of personality: extraversion (E) and neuroticism (N), to which he later added a third dimension called psychoticism (P, risk-taking, impulsiveness) after studying individuals suffering from mental illness (Eysenck and Eysenck, 1976). In parallel, the “Five Factor Model” (FFM), offers another structure organizing the personality traits into five factors (McCrae and John, 1992): Extraversion (E, sociable, outgoing, openly expressive, oriented to the outer world), Agreeableness (A, cooperative, unselfish, compassionate), Conscientiousness (C, organized, responsible, self-disciplined), Neuroticism (N, emotionally unstable, anxious, prone to experience psychological distress and negative emotions) and Openness to experience (O, imaginative, creative, willing to experience new things) (VandenBos, 2007). Lewis Goldberg contributed to this area by developing the Big Five Factor structure and the scales using different samples and factorial analytic methods and proposed the name “Big Five” (Goldberg, 1992; Boudreaux and Ozer, 2015).

Since then, multiple instruments have been developed, used and revised, such as the NEO Personality Inventory (NEO-PI-R) (Costa and McCrae, 1992; McCrae and Costa, 2010), the Big Five Inventory (BFI) (John et al., 1991), the International Personality Item Pool (IPIP) (Goldberg, 1999)– a repository of over 2000 personality items that are used to develop personality inventories-, the Eysenck Personality Questionnaire (EPQ) (Eysenck and Eysenck, 1975) and the Zuckerman-Kuhlman Personality Questionnaire (ZKPQ) (Zuckerman, 2002).

In addition to its primary role in Eysenck’s theory, and its inclusion as a factor in the Big Five, neuroticism is a substantial target in mental health. It is strongly linked to emotional instability and the experience of psychological distress (e.g., anxiety, hostility, depressed mood) (Costa and McCrae, 1992; Diener et al., 2003). These experiences are correlated with low quality of life and low overall satisfaction. Furthermore, evidence also suggests that neuroticism might be related to susceptibility to the development of mental health disorders (e.g., mood and substance disorders) (Kotov et al., 2010), as well as physical illness (e.g., cardiac problems, immune functioning, irritable bowel syndrome) (Lahey, 2009), and the way people interact with and respond to these and other afflictions. Taken together, this indicates that neuroticism has important health implications and a large impact on daily life (Widiger and Oltmanns, 2017).

Mindfulness, based on Buddhist contemplative practices adapted to the Western population, context, and needs, was introduced in research a few decades ago by Kabat-Zinn. He defined mindfulness as “the awareness that emerges through paying attention on purpose, in the present moment, and nonjudgmentally to the unfolding of experience moment by moment” (Kabat-Zinn, 2003). Kabat-Zinn pioneered the development of the Mindfulness-Based Stress Reduction Program (MBSR) designed to treat stress, anxiety, and pain by developing awareness and acceptance of feelings, thoughts, and body sensations through a combination of mindfulness meditation, body awareness, and therapeutic yoga postures (de Vibe et al., 2012). Since then, scientific interest in this practice has grown, and so has the literature exploring its effect on overall health. According to PubMed, in the past year, the term “mindfulness” appeared in the titles of 3,660 articles.

One of the main goals of mindfulness practice is to develop skills and a different relationship with our emotions, thoughts, and experiences. This practice does not aim to eliminate negative experiences, but rather to learn how to approach them with equanimity, non-judgmentally, and in a more flexible way (Kabat-Zinn, 2003). There are two main elements included in the practice of mindfulness: attention with intentionality and the quality thereof. Mindfulness focus is to bring awareness or attention to the present moment, but with specific attitudes, such as non-judgment, openness, acceptance, and curiosity (Baer et al., 2022). Recognizing what is happening from a place of experiential awareness, without trying to change or control anything.

In this sense, mindfulness can be conceptualized as a trait, or dispositional mindfulness, considering that there are some people who tend to display the skills of being mindfully aware in the daily lives without specific mindfulness training or meditation practice (Hart et al., 2013). However, this way of engaging with everyday life, can be trained and the mindful skills can be developed by means of specific interventions and trainings. In the present study, the term “mindfulness” addresses this inherent capacity, while mindfulness-based interventions (MBIs) will be address as such and refers to structured methods designed to teach and reinforce mindfulness skills. On the other hand, the facets of mindfulness are the specific components or variables that make up the overall mindfulness skill and are assessed by questionnaires.

MBIs are used to cultivate and develop this attitudes. They are 6 or 8-week programs that include mindfulness meditation (MM) and encourage home practice of meditation and other components (e.g., yoga practice). In addition to the MBSR program pioneered by Kabbat-Zin to reduce stress, anxiety, and chronic pain, other MBIs, such as Mindfulness-Based Cognitive Therapy (MBCT), have been studied. MBCT, combines MM with cognitive therapy, was adapted from the MBSR to prevent relapse in depression (Segal et al., 2013). Moreover, mindfulness principles are also included in other therapies including Dialectical Behavior Therapy (DBT), used to treat borderline personality disorder (BPD), which is based on cognitive-behavioral therapy (CBT) and the acceptance of feelings and behaviors (Dimeff and Linehan, 2001), or Acceptance and Commitment Therapy (ACT), aimed at increasing psychological flexibility by means of mindfulness and the acceptance of one’s own feelings and thoughts (Hayes et al., 2006).

As interest in mindfulness, MBIs and their applications has grown, so has the need for instruments to empirically assess it. This has allowed for a better understanding of the psychological processes involved and to determine its relationship with other psychological constructs (Baer et al., 2006). In this regard, some of the instruments currently available and commonly used are (Baer et al., 2022): the Mindful Attention Awareness Scale (MAAS) (Brown and Ryan, 2003), the Five Facet Mindfulness Questionnaire (FFMQ) (Baer et al., 2006), the Kentucky Inventory of Mindfulness Skills (KIMS) (Baer et al., 2004), the Freiburg Mindfulness Inventory (FMI) (Buchheld et al., 2001), and the Cognitive Affective Mindfulness Scale—Revised (CAMS–R) (Hayes and Feldman, 2004). Despite their differences, all these questionnaires assess central facets of mindfulness practice, such as acting with awareness (e.g., fully engaging with the present), observing (e.g., noticing internal and external experiences), describing (e.g., being able to express experiences with words), non-judging (e.g., observing experiences without judging them), and non-reactivity (e.g., observing experiences without reacting to them) (Baer et al., 2022).

Given that the main goal of MBIs is to develop abilities to regulate and accept how we relate to our experiences, emotions, and thoughts -particularly negative ones- it is hypothesized that these interventions could reduce the expression and experience of neuroticism.

Previous studies have shed some light on this claim. Giluk (2009) studied the relationship between mindfulness and the Big Five personality traits and found that neuroticism had the strongest correlation with mindfulness. Neuroticism was also positively associated with worry, avoidance, and rumination, and inversely associated with mindfulness. Higher scores of neuroticism and lower scores with the remaining personality traits were associated with maladaptive emotional regulation strategies (Barańczuk, 2019).

Considering neuroticism is one of the most robust higher-order personality traits associated with negative emotionality, where self-awareness is focused on distress and negative emotions, and risk of mental disorders, and mindfulness is a strategy to develop self-awareness based on equanimity, non-judgmentally, and flexibility, we objective of this review was to analyze and discuss the relationship between neuroticism as a personality trait and mindfulness. We first identified the studies according to these terms, selected those following the eligibility criteria, and then organized and summarized the results. We first describe the studies addressing this relationship in a broad sense, then refer to this relationship in more constricted areas such as mental health (depression, anxiety, stress, post-traumatic stress disorder, or other diseases) and cognitive and psychological variables (inflexibility, hardiness, cognitive reappraisal), and finally address the outcomes of those studies, including mindfulness-based interventions.

## 2 Methods

### 2.1 Search strategy

To conduct the present review, we consulted two databases to identify the literature: PubMed and PsycINFO, from inception until December 2023. We used the terms “neuroticism” AND “mindfulness” to identify literature with no restrictions regarding population, date or awareness-based interventions.

Articles for selection were recorded using Rayyan, a web-based data synthesis software program (Ouzzani et al., 2016).

### 2.2 Eligibility criteria

From the search using the terms “neuroticism” AND “mindfulness” the articles were selected according to the following inclusion criteria: (i) to be in English or Spanish and (ii) to contain primary outcome measures that assessed neuroticism and/or mindfulness using validated instruments (e.g., FFMQ, MAAS, NEO-PI, or BFI). Articles were excluded if they were in other languages, described as protocols, dissertations, or validations of instruments, or if the primary outcomes were different from mindfulness and/or neuroticism. No awareness-based interventions were excluded.

### 2.3 Method of synthesis

To summarize the results, we opted for a scoping review that allowed us to organize and synthesize the main findings. Following the recommendations of Aveyard (2014) and other published reviews (McVeigh et al., 2021), we analyzed our articles’ main findings, identified the themes that would allow us to answer our research questions, and to summarize the results, outline the relationship between the evidence, and draw conclusions. The scoping review was conducted by two independent reviewers using pretested forms.

## 3 Results

**Figure 1:**
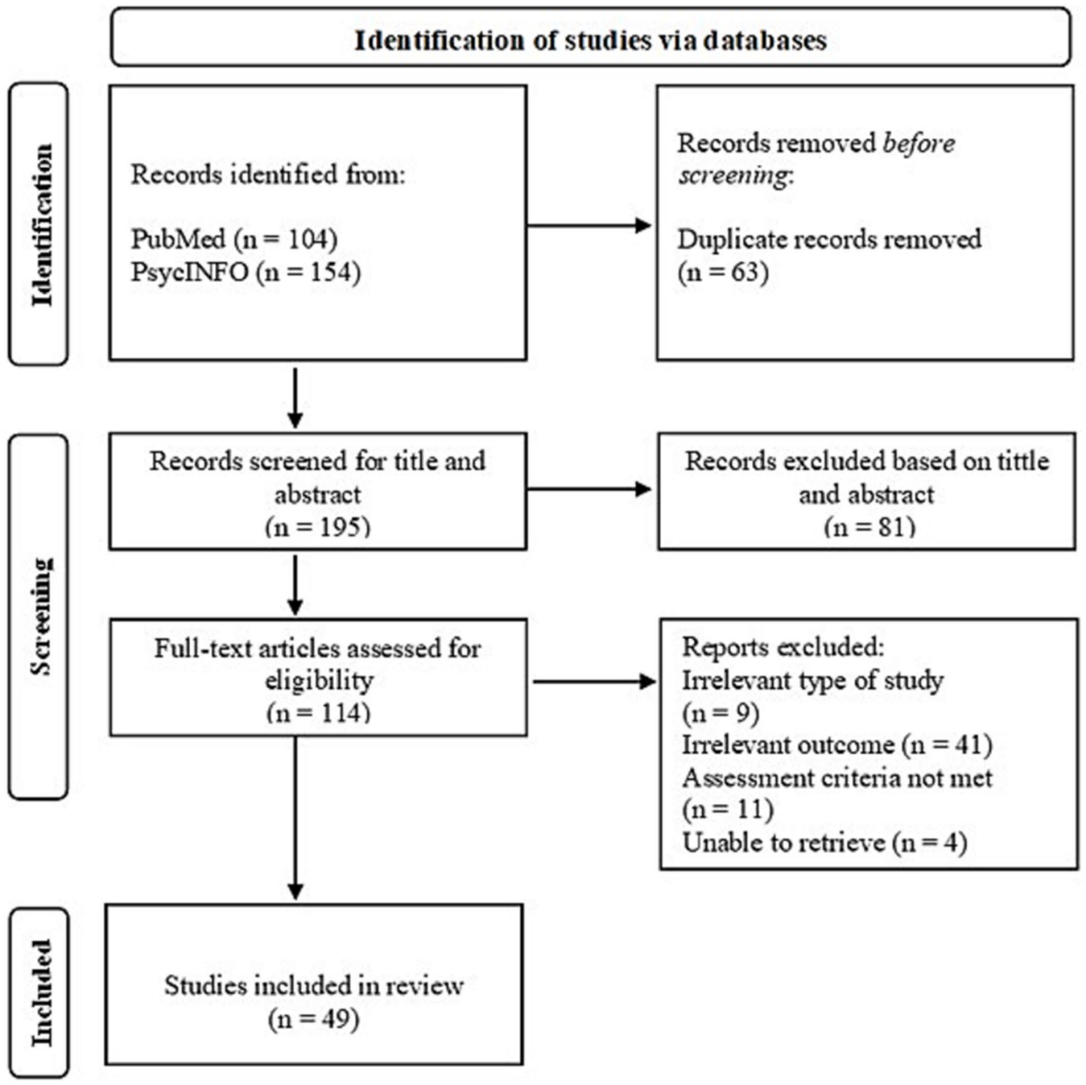
Search strategy flow diagram.

After the search, 258 articles were identified and saved in Rayyan, and all duplicates were removed, 195 articles (Figure 1). First, titles and abstracts were screened, and 81 articles were excluded for various reasons (e.g., different languages, protocols, and validation of instruments). The remaining 114 were selected for full-text review by one author (information removed for anonymized review) to ensure that all eligibility criteria were met. Discrepancies or doubts about the inclusion of articles were discussed with two authors (information removed for anonymized review), and finally, 49 articles were included in this review. For additional information, see the references listed in Table 1 (main characteristics of the participants) and Tables 2–6 (main characteristics of the studies, including conclusions).

**Table 1:** Main characteristics of the sample’s studies.

Author (year)	N, % female	Mean age	Participants
An et al. (2019)	443 (53%)	14.44	Adolescents
Armstrong and Rimes (2016)	34 (91%)	29.55	Under/postgraduate students and staff from a university
Barnhofer et al. (2011)	144 (60%)	43	Volunteers from community
Boelen and Lenferink (2018)	S1: 314 (87.6%) S2: 205 (90.2%)	S1: 21.8 S2: 21.3	University students
Calvete et al. (2020)	571 (50.6%)	14.12	Adolescents
Chen et al. (2023)	260 (76.15%)	19.20	University students
Cillessen et al. (2018)	245 (85.7%)	51.65	Volunteers with cancer diagnosis
Crone et al. (2023)	215 (41.57%)	27.32	University students
de Vibe et al. (2015)	288 (76%)	24	University students
Dixon and Overall (2016)	159 (83.6%)	21.82	Undergraduate students
Drake et al. (2017)	165 (81.8%)	38.69	Volunteers from community
Elices et al. (2015)	133 (88%)	30.46	Outpatients from a borderline personality disorder unit
Elliot et al. (2019)	191 (n/a)	72.4	Volunteers from community
Fabbro et al. (2020)	39 (100%)	50.81	Volunteers from community (teachers)
Feltman et al. (2009)	S1: 195 (59%) S2: 94 (52.1%)	n/a	Undergraduate students
Fetterman et al. (2010)	S1:91 (56%) S2:67 (n/a) S3:98 (58.8%)	n/a	Undergraduate students
Gautam et al. (2019)	801 (68.6%)	19.03	Undergraduate students
Haliwa et al. (2021)	594 (54%)	46.48	Volunteers from community (Amazon’s Mechanical Turk)
Halland et al. (2015)	288 (76%)	23.8	University students
Hanley (2016)	458 (78%)	21	College students
Hanley et al. (2019)	288 (76%)	23.81	University students
Heshmati and Pellerone (2019)	150 (50.7%)	22	College students
Hou et al. (2022)	1,103 (55.3%)	24.32	Postgraduate students
Iani et al. (2017)	211 (72%)	56.4	University students
Jacobs et al. (2011)	60 (53.3%)	48	Volunteers from community
Jagielski et al. (2020)	280 (100%)	54	Volunteers with cancer diagnosis
Karing (2021)	2,548 (74.8%)	23.67	University students
Karl et al. (2021)	331 (71%)	19.34	University students
Kondracki et al. (2021)	1,003 (77.6%)	21.9	College students
Kowalski and Schermer (2019)	258 (60.1%)	19.46	University students
Latzman and Masuda (2013)	429 (79.7%)	21.26	University students
Lee and Bowen (2015)	39 (0%)	38.44	Incarcerated adults with drug abuse disorders
Mather et al. (2019)	229 (n/a)	21	University students and snowball sample
Nyklíček and Irrmischer (2017)	167 (70%)	45.8	Volunteers from community
Oken et al. (2017)	128 (100%)	59.8	Volunteers from community
Palmer et al. (2023)	372 (60.5%)	19.19	Volunteers from community (Amazon’s Mechanical Turk)
Pérez-Yus et al. (2020)	94 (62.7%)	46.77	Volunteers from community
Polizzi et al. (2023)	495 (67.9%)	19.19	University students
Quintana et al. (2017)	80 (90%)	22.4	University students
Smith et al. (2008)	50 (80%)	44.94	A mixed community sample
Spinhoven et al. (2017a)	138 (68.1%)	50.5	A mixed community sample
Spinhoven et al. (2017b)	278 (68.7%)	50.6	A mixed community sample
Thompson and Waltz (2007)	S1: 167 (70.6%) S2: 203 (55.1%)	n/a	University students
Tucker et al. (2014)	315 (64.8%)	19.34	University students
Van den Hurk et al. (2011)	70 (67.1%)	48.35	A mixed community sample
Van Dijk et al. (2015)	S1: 179 (66%) S2: 208 (76.9%)	S1:22 S2:23	University students
Wang et al. (2022)	1,074 (60.7%)	21.12	Undergraduate students
Wupperman et al. (2008)	342 (73%)	n/a	University students
Wupperman et al. (2009)	70 (84.2%)	38.3	Psychiatric inpatients from a trauma unit

**Table 2:** Brief description and main findings of the relationship between mindfulness and neuroticism.

Author (year)	Aim of the study	Design	Measures	Main findings/conclusions
Haliwa et al. (2021)	Associations among mindfulness and all personality traits	Cross-sectional	MAAS, FFMQ, CAMS-R, Mini-IPIP	MF was negatively correlated with N across all scales MAAS *r* = −0.46, CAMS-R *r* = −0.59, FFMQ *r* = −0.63, (*p* < 0.001) Higher MF was related with lower N across all scales (*p* < 0.001)
Hanley (2016)	Relationship between DM and the FFM	Cross-sectional	FFMQ, 44-item BFI	N was correlated with DM (*p* < 0.001) A “self-regulation” (acting with awareness, non-reactivity, and non-judging) cluster was identified
Karl et al. (2021)	Relationship between neuroticism, mindfulness and negative affect.	Cross-sectional	BFI-2, RST-PQ, PANAS, FFMQ-SF	Non-Judgmental Acceptance and Acting with Awareness facets of mindfulness were affected by negative affect change, but not Attention.
Mather et al. (2019)	Relationship between facets of personality and DM	Factorial quantitative	FFMQ, NEO-PI-R	Three MF domains (non-judging, non-reactivity and acting with awareness) loaded negatively to N and showed negative correlations with all N components.
Quintana et al. (2017)	Relationship between mindfulness, personality, and suggestibility	Correlational	FFMQ, NEO-FFI	FFMQ facets (describe act with awareness, non-judging, and non-reactivity) correlated negatively and significantly with N (*p* < 0.001).
Thompson and Waltz (2007)	Relationship between measures of everyday mindfulness and mindfulness during meditation, and between measures of mindfulness and personality characteristics	Quasi-Experimental	MAAS, CAMS-R, TMS, 50-items IPIP	No significant differences between everyday MF and MF during meditation. MF was negatively correlated with N No sig differences between naïve and meditators in MF scores
Van den Hurk et al. (2011)	Relationship between mindfulness meditation and personality traits, and the mediating role of mindfulness skills	Cross-sectional	KIMS, NEO-FFI	No significant differences between the practice and N scores. MM experience was correlated with N (*p* < 0.05)
Wang et al. (2022)	Longitudinal association between mindfulness and N	Longitudinal	MAAS, N scale BFI	Between-person and within-person significant negative and longitudinal association between MF and N (*p* < 0.001)

BFI-2-S, Big Five Inventory-2 Short Form; CAMS-R, Cognitive and Affective Mindfulness Scale-Revised; FFMQ, Five Facet Mindfulness Questionnaire; FFMQ- SF, Five Facet Mindfulness Questionnaire Short Form; IPIP, International Personality Item Pool; KIMS, Kentucky Inventory of Mindfulness Skills; MAAS, Mindful Attention Awareness Scale; MF, mindfulness; MM, mindfulness meditation; N, Neuroticism; NEO-FFI, NEO Five Factory Inventory; NEO-PI-R, Revised NEO Personality Inventory; PANAS, Positive and Negative Affect Schedule; RST-PQ-S, Reinforcement Sensitivity Theory Personality Questionnaire; TMS, Toronto Mindfulness Scale.

**Table 3:** Brief description and main findings of mindfulness and neuroticism in relation with mental health.

Author (year)	Aim of the study	Design	Measures	Main findings/conclusions
An et al. (2019)	Role of DM between N, PTSD and depression following a traumatic event	Cross-sectional	MAAS, N scale NEO-PI-R	Significant correlations between neuroticism, dispositional MF, PTSD, and depression symptoms (*p* < 0.001) MF mediated the relationship between N and PTSD and between N and D
Barnhofer et al. (2011)	Role of DM between N and depressive symptoms.	Longitudinal	FFMQ, EPQ	N correlated with the severity of current D symptoms (*p* < 0.001) and total FFMQ inversely correlated with N and current D symptoms (*p* < 0.001) There was a moderator effect of dispositional MF
Boelen and Lenferink (2018)	Association between acceptance, trait mindfulness, analog PTS symptoms, N, worry and rumination	S1: Cross-sectional S2: Prospective	MAAS, EPQ-R-N	In both samples analog PTS was significantly correlated with more worry, N, rumination, and less acceptance and MF. Experiential acceptances and MF explained analog PTS in S1. In S2, MF was associated with analog PTS after 1 year considering the rest of the variables
Calvete et al. (2020)	Profiles of mindfulness facets in adolescents and their association with emotional and personality variables	Cross-sectional	FFMQ-A-SF, N scale NEO-PI-R	Acting with awareness and non-judging was negatively correlated with D, maladaptive schemes, stress, and N Observing was positively correlated with D, maladaptive schemes, stress and extraversion. Three profiles were identified (1) moderate MF; (2) judgmental observing (lower scores in all facets but observing); (3) non-judgmentally aware (higher scores in acting with awareness and non-judging, lower in observing and non-reactivity)
Dixon and Overall (2016)	Role of DM in negative reactions to day-to-day stressors	Prospective	MAAS, 4-item N subscale Mini-IPIP	MF was negatively correlated with N and depressive symptoms and emotion regulation (*p* < 0.01), and N was positively correlated with depressive symptoms (*p* < 0.01) The association between MF and daily stress was significant
Drake et al. (2017)	Relationship between personality and distress, and the moderator effect of DM	Cross-sectional	FMI-14, 44-item BFI	N was positively correlated with non-specific distress and negatively with MF (*p* < 0.01). N explained 34% of the variance in non-specific distress The relationship between MF and non-specific distress was significantly moderated by mindfulness (*p* = 0.000)
Elices et al. (2015)	Relationship between temperamental traits and childhood maltreatment, and mindfulness in BPD	Cross-sectional	FFMQ, ZKPQ	Negative correlations between sexual abuse and acting with awareness (*p* = 0.03) and non-judging (*p* = 0.01), N with acting with awareness, non-judging, and non-reactivity (*p* < 0.001). N and impulsivity were significant predictors of non-judging and N, and sexual abuse were significant predictors of acting with awareness
Feltman et al. (2009)	Mindfulness as a moderator of N and their relationship with anger and depressive symptoms	Cross-sectional	MAAS, 10-item BFI	N was positively correlated with anger and depressive symptoms and negatively with MF (*p* < 0.01) MF moderated the relationship between N-anger/depressive symptoms
Fetterman et al. (2010)	Relationship between N and behavioral dysregulation, and the role of mindfulness as mediator	Cross-sectional	MAAS, 10-item BFI	N was negatively correlated with MF and self-control and positively with impulsivity; MF was positively correlated with self-control. MF was a significant mediator across all relationships between N and self-control and impulsivity (*p* < 0.01)
Gautam et al. (2019)	Relationships between procrastination, anxiety, and mindfulness	Cross-sectional	FFMQ, NEO-FFI	Non-judging, acting with awareness, describing and non-reactivity were correlated with anxiety and procrastination (*p* < 0.001) Increased anxiety, N and decreased MF were related to increased procrastination
Heshmati and Pellerone (2019)	Relationship between personality traits, DM, and alexithymia	Cross-sectional	FMI-14, NEO-FFI	Alexithymia was negatively correlated with DM and positively with N (*p* < 0.01) N was as strong predictor of alexithymia
Iani et al. (2017)	Mindfulness facets in association with N and well-being	Cross-sectional	FFMQ-SF, 24-item IPIP-NEO	N was positively correlated with anxiety and depression (*p* < 0.01), and negatively correlated with all domains of MF and psychological well-being (*p* < 0.01)
Karing (2021)	Prevalence of anxiety, depression, and stress and association of risk and protective factors	Cross-sectional	MAAS, BFI-S	N, optimism, MF and COVID-19 stressors significantly predicted depression, anxiety, and stress. Higher scores on N predicted higher depression, anxiety, and stress (*p* < 0.001). Higher levels of MF were related to lower depression, anxiety, and stress (*p* < 0.001).
Polizzi et al. (2023)	Relationship between mindfulness facets and COVID19-related stress (CS), considering psychological distress indicators (e.g., N) and social desirability	Cross-sectional	FFMQ, N scale NEO-FFI	N and negative affect correlated positively with CS (*p* ≤ 0.001). CS negatively correlated with some mindfulness facets (*p* ≤ 0.001). Acting with awareness and non-judging were related with less CS, while N was associated with increased CS
Spinhoven et al. (2017b)	Relationship between mindfulness and Big Five and identify which mindfulness facets are strongly associated with personality domains implicated in onset and maintenance of depression	Cross-sectional (subset of data from RCT)	FFMQ, NEO-PI-R	MF scores and non-judging, non-reactivity and acting with awareness (self-regulation factor) were negatively correlated with N (*p* < 0.001) N dimensions were significant in explaining the variance of mindful self-regulation, depression, anxiety and impulsiveness.
Tucker et al. (2014)	Whether the relationship between personality and suicidal ideation are moderated by mindfulness	Cross-sectional	FFMQ, FFF	Suicidal ideation was negatively correlated with MF and extraversion, and positively correlated with N (*p* < 0.01) MF moderated the relationship between N and suicidal ideation
Wupperman et al. (2008)	Whether mindfulness deficits underlie BPD features and areas of dysfunction	Cross-sectional	MAAS, EPQR-A	MF was positively correlated with interpersonal effectiveness, and negatively correlated with BPD features, impulsive emotional regulation and N MF predicted BPD features even when all other variables were controlled
Wupperman et al. (2009)	Whether mindfulness deficits predict BPD features and related behavioral dysfunction	Cross-sectional	MAAS, EPQR-A	MF was positively correlated with interpersonal effectiveness, and negatively correlated with BPD features, impulsive emotion regulation and N MF predicted BPD features even when all other variables were controlled

BFI, Big Five Inventory; BFI-S, German Short Version Big Five Inventory; BPD, Borderline Personality Disorder; D, Depression/Depressive; EPQ, Eysenck Personality Questionnaire; EPQR-A, Eysenck Personality Questionnaire-Revised Abbreviated; FFF, Five Factor Form; FFM, Five Factor Model of personality; FFMQ, Five Facet Mindfulness Questionnaire; FFMQ-A-SF, Five Facet Mindfulness Questionnaire-Adolescents-Short Form; IPIP, International Personality Item Pool; MAAS, Mindful Attention Awareness Scale; MF, mindfulness; N, Neuroticism; NEO-FFI, NEO Five Factory Inventory; NEO-PI-R, Revised NEO Personality Inventory; PTS, post-traumatic stress; S, Sample; ZKPQ, Zuckerman-Kuhlman Personality Questionnaire.

**Table 4:** Brief description and main findings of mindfulness and neuroticism in relation with cognitive outcomes.

Author (year)	Aim of the study	Design	Measures	Main findings/conclusions
Chen et al. (2023)	Role of mindfulness and cognitive bias in emotion regulation in neurotic individuals	Cross-sectional	FFMQ, EPQ-RSC	N correlated positively with negative cognitive bias and emotion regulation, and positively MF (*p* < 0.001) High neuroticism participants show negative attention, memory, and interpretation biases
Hou et al. (2022)	Whether DM mediated the relationship between N and depression, and the role of cognitive reappraisal	Cross-sectional	MAAS, N scale NEO-FFI	N was positively correlated with D and negatively correlated with DM and cognitive reappraisal (*p* < 0.001) DM mediated the relationship between N and depression, and cognitive reappraisal moderated the effect
Kondracki et al. (2021)	Relationship between N, mindfulness, and cognitive failures	Cross-sectional	FFMQ, EPQ-R-S	N was positively correlated with cognitive failures (only in females, *p* < 0.001), and negatively with MF (both sexes, *p* < 0.001). MF correlated negatively with cognitive failures (both sexes, *p* < 0.001). MF mediated the relationship between N and cognitive failures
Kowalski and Schermer (2019)	Relationship between hardiness, mindfulness, rumination, worry, anxiety, N, and health	Cross-sectional	FFMQ, 20-N items IPIP	Hardiness was negatively correlated with rumination, worry, anxiety, and N; and positively correlated with MF and health outcomes (*p* < 0.001)
Latzman and Masuda (2013)	Associations between physiological inflexibility, mindfulness and the Big Five	Cross-sectional	MAAS, 44-item BFI	MF was negatively correlated with psychological inflexibility and N; N was positively correlated with psychological inflexibility. All personality dimensions contributed to explaining psychological inflexibility, but N showed the strongest association (*p* < 0.001)
Pérez-Yus et al. (2020)	Variables associated with effectiveness in negotiation and the role of mindfulness	Cross-sectional	FFMQ-SF, NEO-FFI	Effectiveness in negotiation was correlated with age, emotional intelligence, MF, personality (E, O, C), motivation, style of negotiation and negatively correlated with N Meditators displayed more clarity, MF, and were less N, and more effective at negotiating

BFI, Big Five Inventory; DM, dispositional mindfulness; EPQ-R-S, Eysenck Personality Questionnaire-Revised Short Scale; EPQ-RSC, Eysenck Personality Questionnaire-Revised Short Scale for Chinese; FFMQ, Five Facet Mindfulness Questionnaire; FFMQ- SF, Five Facet Mindfulness Questionnaire Short Form; IPIP, International Personality Item Pool; MAAS, Mindful Attention Awareness Scale; MF, mindfulness; N, Neuroticism; NEO-FFI, NEO Five Factory Inven.

**Table 5:** Brief description and main findings of studies evaluating MBSR.

Author (year)	Aim of the study	Intervention	Measures	Main findings/conclusions
de Vibe et al. (2015)	Whether personality factors and baseline mindfulness moderated the effect of MBSR on mental distress, study stress and subjective well-being	MBSR vs. No active control	FFMQ, BCI	Higher N scores at baseline showed a larger effect on mental distress (*p* = 0.05) and study stress (*p* = 0.01). MBSR lowered mental distress and improved subjective well-being as N increased
Elliot et al. (2019)	Interaction between mindfulness and N, and whether the effects of MBSR depend on the levels of N (baseline scores)	MBSR vs. Wait-list control	MAAS, N scale NEO-FFI	Higher MF scores were associated with less D, negative affect and physical symptoms, and better sleep quality. High MF and less D was only significant at high levels of N
Halland et al. (2015)	Effect of mindfulness training on the use of coping strategies and the moderator effect of personality	MBSR vs. No active control	BCI	N was positively correlated with avoidance-focused coping, and negatively correlated with seeking social support and problem-focused coping (*p* < 0.001) Participants in MBSR used more problem-focused coping (*p* = 0.01) N moderated the effect of MBSR on the use of avoidance-focused coping and seeking social support
Hanley et al. (2019)	Effect of mindfulness training on N and psychological distress	MBSR vs. No active control	BCI	Significant intervention x time interaction effect on N (*p* = 0.041, *d* = 0.28) and psychological distress (*p* = 0.001, *d* = 0.21). Mediation analysis reported that the relationship between MBSR and psychological distress was mediated by N (*b* = −0.09, *p* = 0.020, 95% CI = −1.649, −0.304).
Jagielski et al. (2020)	Whether personality factors accounted for variability in response to MBSR	MBSR vs. TAU	NEO-PI-R	There was a significant effect of treatment on lower conscientiousness and high N at 12-month follow-up, showing less distress
Nyklíček and Irrmischer (2017)	Moderator effect of personality regarding changes of mood associated with MBSR	MBSR (no comparator group)	NEO-PI-R	Higher N scores showed decreases in anxiety and depressed mood (*p* < 0.001)
Smith et al. (2008)	Compare the effects of MBSR and CBSR on perceived stress and depression	MBSR vs. CBSR	MAAS, N scale BFI	There were sig. Pre-post changes in the MBSR group with an increase in mindfulness and well-being and decreases in perceived stress, D, and N (*p* = 0.000)
Van Dijk et al. (2015)	Interest in participating in a MBSR training, and the differences between interested and non-interested subjects and participants and non-participants in the training	MBSR	FFMQ, NEO-FFI	The interested population reported higher levels of psychological distress (*p* = 0.004) and neuroticism (*p* < 0.001) Participants in the RCT reported higher baseline psychological distress (*p* = 0.001) and less mindfulness skills (*p* = 0.002)

BCI, Basic Character Inventory; BFI, Big Five Inventory; D, Depression/Depressive; FFMQ, Five Facet Mindfulness Questionnaire; MAAS, Mindful Attention Awareness Scale; MBSR, Mindfulness-based Stress Reduction; MF, mindfulness.

**Table 6:** Brief description and main findings of the effects of other mindfulness-based interventions.

Author (year)	Aim of the study	Intervention	Measures	Main findings/conclusions
Armstrong and Rimes (2016)	Efficacy of MBCT in individuals with high N	MBCT vs. Online self-help	FFMQ, EPQ-R-N	The MBCT group had lower levels of N post-treatment (*p* = 0.003), lower levels of rumination (*p* = 0.016), higher levels of self-compassion (*p* = 0.001), and decentering (*p* = 0.006). There were no sig. Differences between groups in anxiety or depression
Cillessen et al. (2018)	Long-term effect of e/MBCT in cancer patients	MBCT vs. eMBCT	FFMQ-SF, NEO-FFI	Psychological distress and rumination decreased (*p* = 0.001), and positive mental health and mental health related QoL increased (*p* = 0.001), in both groups at follow-up
Spinhoven et al. (2017a)	Whether changes in mindfulness skills after MBCT predict long term changes in personality traits	MBCT	FFMQ, NEO-PI-R	There were sig. Changes in N scores (*p* = 0.001) with a moderate reduction (*d* = 0.54). There was a sig. Effect of time for overall FFMQ (*p* = 0.001). Changes in total FFMQ predicted a 5.0% additional variance in changes in N, the subscale acting with awareness was the only sig. Unique predictor of changes in N (*p* = 0.001).
Crone et al. (2023)	Effect of a mindfulness-based training program on emotional well-being	MF-based training vs. Wait-list control	MAAS, FFMQ-SF, TIPI	The intervention reduced N compared with control group (*p* < 0.05) in both phases. Changes in mindfulness were observed in all facets in the intervention group during phase 2 (*p* < 0.05; *p* < 0.01)
Fabbro et al. (2020)	Effects of MM on personality traits and perceived stress and burn-out	MM vs. Wait-list control	FFMQ, 44-item BFI	The MM group displayed higher DM (*p* = 0.004), a decrease in N (*p* < 0.001) and in the perception of stress (*p* = 0.010)
Jacobs et al. (2011)	Effect of a meditation retreat on mindfulness, purpose in life, perceived control, N, and immune cell telomerase activity	3-month meditation retreat vs. Wait-list control	FFMQ, N scale BFI	There was a significant increase in mindfulness and a decrease in N in the retreat group (*p* < 0.0001). Participants reporting less psychological functioning were the ones showing greater improvements post-intervention
Lee and Bowen (2015)	Relationship between the Big Five and mindfulness and the response to a meditation practice	MF-based training (no comparator group)	TMS, NEO-FFI	Results contradicted the hypothesis. N was positively correlated with MF, and there were no post-intervention differences in curiosity and in decentering.
Oken et al. (2017)	Mindfulness meditation improves cognition and mental health and physiology	MM vs. Wait-list control	MAAS, KIMS, NEO-FFI	MM intervention decreased scores in negative affect, D, N, stress, and improved mental health outcomes
Palmer et al. (2023)	Dose–response relationship and moderators of the effect of a single-session mindfulness meditation session	10 min meditation vs. 20 min meditation vs. 10 min control vs. 20 min control	CAMS-R, SMS, N scale of BFI	Independent of interventions all participants sowed increased state mindfulness, decreased state anxiety, negative and positive affect. N did not moderate the effect of any of the interventions.

BFI, Big Five Inventory; CAMS-R, Cognitive and Affective Mindfulness Scale-Revised; DM, dispositional mindfulness; EPQ-R-N, Eysenck Personality Questionnaire-Revised-Neuroticism scale; FFMQ, Five Facet Mindfulness Questionnaire; FFMQ- SF, Five Facet Mindfulness Questionnaire Short Form; KIMS, Kentucky Inventory of Mindfulness Skills; MAAS, Mindful Attention Awareness Scale; MM, mindfulness meditation; N, Neuroticism; NEO-FFI, NEO Five Factory Inventory; NEO-PI-R, Revised NEO Personality Inventory; SMS, State Mindfulness Scale; TIPI, Ten Item Personality Inventory; TMS, Toronto Mindfulness Scale.

### 3.1 Characteristics of the studies included

The 49 studies selected for the present review (see Table 1) took place in different countries (Australia, Canada, China, Iran, Italy, Denmark, Germany, the Netherlands, New Zealand, Norway, Spain, Taiwan, the UK, and the USA), resulting in a total sample drawn from several different cultures. The sample sizes of the studies ranged from 34 to 2,548 participants (*n* = 17,451), with a mean age of 32.25 years (range from 14.44 to 72.4 years). A percentage of 81.11 of the sample was women, except for one study that included an all-male sample (Lee and Bowen, 2015). The samples comprised students, volunteers from the community, or mixed volunteers from the community with a specific pathology and adolescents. Most of the included studies used a cross-sectional design, while others were randomized controlled trials, longitudinal studies, quasi-experimental studies or other types (the design of each study is indicated in Table 2).

The final 49 studies were grouped and presented over the following sections, starting with the relationship between neuroticism and mindfulness reported in the studies that assessed neuroticism and/or mindfulness using validated instruments. Next, we describe the relationship of these two dimensions and the mindfulness facets with mental health; more specifically with depression, anxiety, posttraumatic stress syndrome and borderline personality disorder. The relationship of mindfulness and neuroticism have been also studied in regard with cognitive impairments; these results are presented in the next section. The last two sections refer to those studies evaluating the effects of Mindfulness-Based Stress Reduction Program (MBSR), and the other mindfulness-based interventions used in the studies included (MBIs, section five).

### 3.2 Relationship between neuroticism and mindfulness

Although all eight studies (Table 2) used different sets of instruments, similar results reported a significant negative correlation, indicating that high mindfulness was associated with low neuroticism (Quintana et al., 2017). When comparing the neuroticism scores between meditators and non-meditators, even if there were no significant differences between the two groups, more experience on meditation was negatively correlated with neuroticism, and this relationship was mediated by the mindfulness facets “acting with awareness” and “non-judging” (van den Hurk et al., 2011). Accordingly, when assessing neuroticism and mindfulness at 4-time points over a year in a sample of undergraduate students, mindfulness was found to be negatively correlated with neuroticism (Wang et al., 2022).

Mindfulness states, particularly emphasizing facets such as self-awareness (“Acting with Awareness”) and emotional processing (“Non-Judgmental Acceptance”) seem to be linked to negative affect and underscored that personality traits and behavioral inhibition did not affect nor predict changes in emotional affect after the exposure to a negative stimulus. The authors posited that a potential constraint lay in the contextual backdrop of the study, coinciding with the onset of the COVID-19 pandemic. They propose that participants, undergraduate students, may have already experienced elevated initial stress levels, potentially masking the underlying connections between personality traits and negative affect (Karl et al., 2021).

Mather et al. (2019) reported negative correlations of three mindfulness facets, non-judging, non-reactivity, and acting with awareness, with all neuroticism components (anxiety, anger, hostility, depression, impulsiveness, vulnerability, and self-consciousness). Consistently, Hanley (2016) outlined the “self-regulation cluster,” which is composed of self-regulation skills associated with three mindfulness facets (non-judging, non-reactivity and acting with awareness) that are negatively correlated with neuroticism. Another study examining the association of mindfulness facets with specific neuroticism aspects (anxiety, anger, depression, self-consciousness, immoderation, and vulnerability) reported that three mindfulness facets were negatively correlated with anxiety, depression and vulnerability, but not with the other neuroticism components. Moreover, the facets observing and describing showed no relationship with anxiety and depression, which aligns with other work showing no relationship between observing and broad neuroticism (Iani et al., 2017).

Furthermore, an investigation of the relationship between the Five-Factor Model and everyday mindfulness (e.g., being mindful during everyday activities) and mindfulness during meditation (e.g., formal meditation practice) revealed that there were no significant differences between the types of mindfulness (Thompson and Waltz, 2007). Haliwa et al. (2021) assessed mindfulness using multiple questionnaires (FFMQ, CAMS-R, and MAAS) and found that all mindfulness measures were negatively correlated with neuroticism, thus strengthening the evidence for this relationship.

### 3.3 Mindfulness and neuroticism in relation with mental health

Iani et al. (2017) assessed the dimensions of mindfulness and their relationship with psychological well-being and neuroticism and found that neuroticism had a positive correlation with depression and anxiety, while a negative relationship with all facets of mindfulness (except for the “observe” and “describe” facets) and psychological well-being. In adolescents, acting with awareness and non-judging was negatively correlated with depression, maladaptive schemes, stress, and neuroticism (Calvete et al., 2020). More specifically, the authors identified a profile of adolescents that exhibited the lowest scores in non-judgment and acting with awareness and who experienced higher levels of depressive symptoms, maladaptive schemes, stress, and neuroticism; as a result, their psychobiological adjustment was poorer among older adolescents. Another profile of adolescents displayed higher scores in acting with awareness and non-judging, and lower scores in observing and non-reactivity; as a result, they exhibited better psychobiological adjustment, characterized by fewer maladaptive schemes, less stress, and less neuroticism.

When Gautam et al. (2019) assessed the presence of anxiety and distress, similar results were obtained. Increased anxiety and neuroticism and decreased mindfulness scores were related to increased procrastination among undergraduate students. Furthermore, Drake et al. (2017) found that non-specific psychological distress was positively correlated with neuroticism and a negatively correlated with mindfulness, which predicted the presence of non-specific distress. Likewise, Dixon and Overall (2016) found that university students who were more “mindful” reported less depressed mood scores even on stressful days, and that daily stress seemed to have a greater impact on depressed mood when individuals scored high on neuroticism, depressive symptoms, and emotional regulation difficulties.

Several studies have confirmed a positive correlation between neuroticism and depressive symptoms and a negative correlation between neuroticism and mindfulness (Feltman et al., 2009; Barnhofer et al., 2011). Neuroticism was also a predictor of depressive symptoms in a study of adolescents who had experienced a tornado (An et al., 2019), and a predictor of depression, anxiety, and stress in a sample of university students (Karing, 2021). In contrast, mindfulness predicted an opposite relationship with depression, anxiety, and stress during the first COVID-19 lockdown in a later study (Karing, 2021). Additionally, neuroticism scores assessed 6 years before assessing depressive symptoms correlated with the severity of current depressive symptoms in a longitudinal cohort study (Barnhofer et al., 2011). It should be noted that in this study, the predictive value of neuroticism on depressive scores was significant when participants displayed low and moderate mindfulness scores. Similarly, Feltman et al. (2009) reported that higher levels of depression were present in a sample that displayed higher levels of neuroticism and less mindfulness, while a study assessing more severe depressive symptoms in a sample of university students reported a positive correlation between the appearance of suicidal ideation and neuroticism and a negative correlation between suicidal ideation and mindfulness (Tucker et al., 2014).

In a cross-sectional study of participants with recurrent depression in remission, which assessed the mindfulness dimensions associated with personality domains and implicated in the relapse of depression, Spinhoven et al. (2017a,b) reported that non-judging, non-reactivity, and acting with awareness skills, described as the “self-regulation factor,” correlated with less expression of neuroticism in terms of depression, anxiety, and impulsiveness.

Regarding trauma-related outcomes, neuroticism was a significant predictor of post-traumatic stress disorder (PTSD) in adolescents who had experienced a tornado (An et al., 2019). Boelen and Lenferink (2018) evaluated the association between experiential acceptance, mindfulness and “analog PTS”—understood as post-traumatic stress associated with negative life events (e.g., mental/physical illness of others, relationship break up, serious interpersonal conflict…), but not compliant with formal PTSD criteria—and their relationship with neuroticism in university students. They found positive correlations between neuroticism and analog PTS, worry, and rumination, and a negative correlation with mindfulness.

On the other hand, Polizzi et al. (2023) assessed the impact of a mass traumatic event and the experience of COVID-19-related stress (CS). Their results indicated that neuroticism was a significant factor explaining CS and that certain mindfulness facets, such as acting with awareness and non-judging, may play an important role in reducing or preventing stress responses.

In contrast, Elices et al. (2015), assessed the relationship between mindfulness, neuroticism, and childhood maltreatment in a sample of outpatients from a borderline personality disorder (BPD) unit, and found that neuroticism-anxiety was negatively correlated with acting with awareness, non-judging, and non-reactivity. This negative association indicated that neuroticism and impulsive sensation seeking were predictors of non-judging, and that neuroticism was also predictive of acting with awareness. In the same study, sexual abuse was correlated with mindfulness deficits, with a negative impact on acting with awareness, and difficulties being present-oriented instead of being more judgmental-oriented, which is a characteristic of BPD.

Similarly, Wupperman et al. (2008, 2009) assessed the relationship between mindfulness deficits and BPD features (e.g., difficulties with emotional regulation, interpersonal effectiveness, and impulsivity). First, they evaluated a student sample without BPD and found that mindfulness was positively correlated with interpersonal effectiveness and negatively correlated with impulsivity, emotional regulation, and neuroticism. Subsequently, they evaluated a sample of psychiatric inpatients and found similar results, confirming that mindfulness deficits are predictors of the expression of BPD (Wupperman et al., 2008).

Assessing behavioral regulation, a related outcome, Fetterman et al. (2010), found that in undergraduate students, neuroticism was negatively correlated with behavioral regulation variables such as self-control, and positively correlated with impulsivity, while higher mindfulness predicted less impulsivity and neuroticism, and more self-control. In relation with this, a core component of successful emotional regulation is the ability to identify and describe our feelings, in contrast with the inability to process emotional information and difficulties in emotional regulation which is known as alexithymia. Related to this, Heshmati and Pellerone (2019) found that alexithymia correlated negatively with dispositional mindfulness and positively with neuroticism and that neuroticism predicted the presence of alexithymia. Additional details can be found on Table 3.

### 3.4 Mindfulness and neuroticism in relation with cognitive outcomes

Latzman and Masuda (2013) evaluated students to study the relationship between mindfulness and psychological inflexibility, understood as a rigid psychological reaction characterized by experiential avoidance and diminished daily function, and associated with depression, anxiety, and general distress. The results indicated that participants who scored higher on neuroticism displayed greater psychological inflexibility, whereas those with higher mindfulness scores displayed less psychological inflexibility.

Kowalski and Schermer (2019) assessed hardiness, characteristic of individuals who remain healthy under stressful conditions. Hardiness is characterized by the belief that one can influence events, commitment to activities, and a tendency to alter the cognitive appraisal of stressful events from negative to positive, viewing them as challenges to be overcome. The authors found that hardiness was negatively correlated with rumination, worry, anxiety, and neuroticism and positively correlated with mindfulness and health outcomes. However, when neuroticism was controlled for the analyses, the relationship between hardiness, rumination, and health was not significant, and the level of significance of the correlations between hardiness, mindfulness, anxiety, and worry was attenuated.

Additionally, Kondracki et al. (2021) reported in that college students that higher neuroticism scores were associated with lower mindfulness and higher everyday cognitive failures. In line with this, Hou et al. (2022), found that neuroticism was positively correlated with depression and negatively with dispositional mindfulness and cognitive reappraisal in postgraduate students. Chen et al. (2023) found that the level of neuroticism exhibited a positive correlation with negative emotion regulation. Negative cognitive bias served as a mediator in the relationship between neuroticism and emotion regulation, while mindfulness played a mediating role in the relationship with negative cognitive bias, forming a sequential connection.

Pérez-Yus et al. (2020) evaluated the role of mindfulness and its association with negotiation effectiveness in an adult sample and found that negotiation effectiveness was positively correlated with age, emotional intelligence, mindfulness, several personality domains (E, O, and C), motivation, and style of negotiation, whereas it was negatively correlated with neuroticism. Furthermore, the results indicated that people who meditated more displayed increased clarity (emotional intelligence), greater mindfulness, less neuroticism, and greater effectiveness in negotiation. Additional information can be found in Table 4.

### 3.5 Evidence from studies evaluating MBSR

MBSR is the most used intervention. In university samples, the results indicated positive effects; MBSR lowered mental distress and study stress and improved subjective well-being as baseline neuroticism increased (de Vibe et al., 2015). Similarly, Halland et al. (2015) reported that following a MBSR intervention, students used more problem-focused coping, and those displaying higher neuroticism scores reduced avoidance coping and increased seeking social support. Moreover, mindfulness training decreased neuroticism and psychological distress during the 6-year follow-up period, and this decrease was associated with reduced psychological distress at the 6-year follow-up (Hanley et al., 2019). Finally, Van Dijk et al. (2015) assessed the interest in participating in a MBSR training and found that interested participants reported higher levels of psychological distress and neuroticism. Furthermore, those who participated displayed lower baseline mindfulness skills compared to non-participants in the training, which could be explained by sampling bias.

The effects of training were also tested in volunteers from the community, and the results were similar. Nyklíček and Irrmischer (2017) assessed the effect of personality on mood changes after MBSR training and found that neuroticism was associated with benefits3 in the reduction of anxiety and depressed mood. Similarly, Smith et al. (2008) compared MBSR with an intervention based on cognitive behavioral stress reduction and found that those participants in the MBSR group displayed higher scores on mindfulness and well-being, and a reduction in perceived stress, depression, and neuroticism than those in the other group. A similar pattern was reported by Jagielski et al. (2020) in a sample of women with breast cancer; the results after the MBSR training indicated that women displaying low conscientiousness (e.g., being relaxed, adaptable, spontaneous) and high neuroticism, reported less distress at 12-month follow-up.

Finally, Elliot et al. (2019), who reported baseline scores before the intervention—found that higher scores of mindfulness were associated with fewer depressive symptoms, negative affect, physical symptoms, and better sleep quality; however, the association of higher mindfulness with fewer depressive symptoms was only significant when the participants displayed higher levels of neuroticism, and neuroticism did not interact with the rest of the outcomes. Additional details have been summarized in Table 5.

### 3.6 Effects of other mindfulness-based interventions

Armstrong and Rimes (2016) used MBCT to compare its efficacy with an online self-help intervention in a sample of university students and staff with high neuroticism. Participants in the MBCT group reported significantly lower levels of neuroticism and rumination, as well as higher levels of self-compassion and decentering (the ability to observe thoughts and feelings as passing mental events) than participants in the online self-help group. There were no changes in levels of depression or anxiety. Another study examining the long-term effects of MBCT compared to internet-based MBCT in a sample of distressed cancer patients indicated that less psychological distress, rumination, and neuroticism at baseline predicted less psychological distress at follow-up in both interventions. The intervention also improved the mental health-related quality of life and positive mental health (Cillessen et al., 2018). Finally, Spinhoven et al. (2017b) analyzed whether MBCT predicted long-term changes in personality and found a significant reduction in neuroticism scores. Additionally, improvements in mindfulness skills predicted changes in neuroticism, and acting with awareness was associated with these changes.

Other nonstandard mindfulness-based interventions. The impact of a single session of mindfulness meditation, regardless of its duration, enhanced state mindfulness. Surprisingly, neuroticism does not mediate these effects (Palmer et al., 2023).

Another study explored the effects of adapted mindfulness training on the well-being of graduate students and reported that the intervention caused a significant reduction in neuroticism, while the effect on mindfulness facets was found only in a subsample (Crone et al., 2023).

Participants trained in mindfulness-oriented meditation (MOM) (based on MBSR, sessions included a 30 min discussion followed by 30 min MOM meditation) reported higher mindfulness scores and decreased neuroticism scores and burnout levels vs. participants on a waiting list in an all-female sample of teachers (Fabbro et al., 2020). By contrast, Lee and Bowen (2015) tested a mindfulness-based training program in an all-male sample of incarcerated men receiving drug abuse treatment. The treatment content included the core components of mindfulness, relapse prevention, and a balanced lifestyle. However, contrary to their own hypothesis, the post-intervention results indicated lower levels of curiosity and decentering and revealed an unexpected positive correlation between mindfulness and neuroticism.

Oken et al. (2017) assessed the effects of mindfulness meditation (MM) vs. a waitlist control in a sample of mildly stressed adults. The intervention lasted 6 weeks and was carried out one-on-one, in contrast to the typical group setting. The results indicated no change in cognitive measures, sleep, positive affect, physiological outcomes (e.g., salivary cortisol, health rate), or, surprisingly, mindfulness measures. However, MM improved negative affect, stress, mental health component, and self-efficacy.

Finally, Jacobs et al. (2011) conducted an experiment that compared a 3-month meditation retreat with a waitlist control to test its effects on telomerase activity, stress, and neuroticism. The intervention was intense and was conducted in an isolated retreat setting; it included two daily group-guided meditations followed by a mean of 6 h solitary meditation during the day and had a weekly individual meeting with an experienced practitioner. The authors reported a significant increase in mindfulness, perceived control, and telomerase activity (measured after treatment), and a decrease in neuroticism in the retreat group compared to the waitlist control group. Additionally, participants who reported less favorable psychological functioning at baseline showed greater improvements post-intervention. Complementary information can be found in Table 6.

## 4 Discussion

Neuroticism, a well-known personality trait with established negative effects on well-being and relationships with several mental, physiological, or cognitive health problems (Lahey, 2009). Meanwhile, the past decade has provided plenty of evidence on beneficial the effects of mindfulness on health-related outcomes (Tomlinson et al., 2017) and mindfulness-based interventions (MBIs) on psychiatric disorders (Goldberg et al., 2018). Our main goal was to review the relationship and the interaction between mindfulness and neuroticism.

### 4.1 Relationship between neuroticism and mindfulness

Consistent findings across various studies indicate that when neuroticism scores are high, mindfulness scores tend to be low, and vice versa. This relationship has been corroborated across different populations of all ages and in several countries. Neuroticism and mindfulness are related to diverse mental health outcomes. Neuroticism shares core characteristics with anxiety and depressive symptoms (Jylhä and Isometsä, 2006) and is a predictor of mental health symptomatology (Newton-Howes et al., 2015). Studies related to emotional and behavioral regulation and psychological inflexibility, once again common characteristics of neuroticism and core components of several mental health problems, are also supported by previous evidence (Paulus et al., 2016).

Giluk (2009) reported that neuroticism is the personality trait most strongly related with mindfulness. Subsequent studies have identified a common pattern of high scores in facets such as “acting with awareness,” “non-judging,” and “non-reactivity,” which have been identified as the “self-regulation cluster” (Hanley, 2016) or “unbiased awareness” (Elices et al., 2015; Spinhoven et al., 2017a,b; Mather et al., 2019; Calvete et al., 2020). These “clusters” have been linked to improved physical and psychological health and coping strategies (Beaulieu et al., 2022; Zhu et al., 2020). However, the possibility that this negative relationship may in all circumstances cannot be ruled out. Since some authors have reported an unexpected positive correlation between mindfulness and neuroticism, further work needs to be done in larger samples to improve understanding of these relationships, both in broad terms and specifically between the mindfulness dimensions and neuroticism components.

Most characteristics of neuroticism correlate with the expression of, predict, or worsen diverse mental and physical health problems. MBIs have shown that training core components can help alleviate or improve symptoms; if not, they can help people suffering from these conditions to manage them or cope with them more adaptively.

### 4.2 Impact on mental health outcomes

Additionally, we assessed the impact of MBIs on other health outcomes (e.g., depression, anxiety, cognitive outcomes). Despite the heterogeneity, the results were mainly consistent across the outcomes.

MBIs were useful for improving symptom management and stress-related disease outcomes in different patient populations, suggesting that these benefits can come from increasing stress resilience pathways in the brain and regulating stress reactivity (hypothalamic pituitary adrenal and sympathetic adrenal medullary axis) (Creswell et al., 2019). Both dispositional and trained mindfulness, can improve patient-reported symptoms across different diagnoses. It is becoming clear that the benefit of mindfulness training comes from the acquisition of coping abilities to deal with ailments, including making conscious healthy decisions and reducing habitual reactions (Greeson and Chin, 2019).

Studies have demonstrated that MBIs significantly affect alexithymia, enhancing awareness of emotions, curiosity about inner experiences, and connection with one’s thoughts and feelings (Norman et al., 2019). Evidence, although limited, indicates that MBIs are promising for treating trauma-related outcomes, such as PTSD, helping individuals tolerate their physical and emotional distress and reduce their hyperarousal symptoms (Niles et al., 2018). Similarly, MBI have proven effective in reducing depression and anxiety among individuals diagnosed with anxiety disorders (Sevilla-Llewellyn-Jones et al., 2018). A Meta-Analytic review with 998 participants examined the effects of MBIs on biomarkers in psychiatric illness, results showed low but significant effects on health status related to biomarkers of low-grade inflammation (Sanada et al., 2020).

Recent studies have explored the long-term effects of mindfulness on mental health, revealing outcomes in areas such as depression, anxiety, and stress management. For instance, a study evaluated the -short, −medium and long-term efficacy of a combined mindfulness intervention (face-to-face intervention plus app), a face-to-face mindfulness intervention alone, and a mindfulness app alone, compared with an active control group (communication training) during the COVID-19 pandemic. The results indicated improvements in mindfulness, emotional regulation and attentional skills up to 12 months after the intervention. In contrast, no significant differences were found between the groups on measures of stress or mental health (anxiety or depression) in the long term. But both the intervention groups and the active control group improved in anxiety in the short and medium term. For depression, only a significant time effect was observed4 months after the intervention. In addition, it was observed that greater use of the mindfulness app could negatively affect stress (Karing, 2024). Another study indicates that a short-term MBI and active control group (relaxation training) improved trait mindfulness and psychological well-being compared with an inactive wait-list group. However, no group differences were found on any of the other variables like decentering depression, anxiety, executive attention, and coping style (Josefsson et al., 2014). A systematic review suggests that MBI’s was effective in reducing stress in the short term, but not in the medium or long term, showing a small effect on stress reduction over a 3-month period. Nevertheless, no significant evidence was found for stress reduction from 3 months onwards (Sosa-Cordobés et al., 2022). This indicates that other strategies, such as ongoing booster sessions, are needed to maintain improvements in the long term.

### 4.3 Impact on cognitive health outcomes

Research supports the benefits of MBIs not only on emotional and behavioral regulation and psychological inflexibility, common features of neuroticism, but also that mindfulness emerges as a significant moderator in the relationship between self-control and psychological symptoms. This suggests that a mindful approach complements self-discipline in improving mental health (Bowlin and Baer, 2012).

In this respect, greater degree of mindful awareness may buffer the effects of psychological inflexibility on distress variables, particularly somatization and anxiety (Masuda et al., 2022). For example, a brief-MBI integrated into a school curriculum enhanced children’s socio-emotional and academic development. Positive changes in dispositional mindfulness led to reductions anxiety and psychological inflexibility. The authors discuss that dispositional mindfulness and emotion regulation work as processes of change that underlie the intervention’s impact (García-Rubio et al., 2023). A longer intervention improved executive functioning measures related to susceptibility to cognitive interference and working memory in high school students (Frank et al., 2021).

Both MBIs and CBT, along with Acceptance and Commitment Therapy (ACT), emphasize that people should focus on the way they relate to their symptoms or thoughts instead of trying to eliminate them, without identifying with them and in a kind manner. They are thus grouped under what are known as third-wave cognitive-behavioral therapies (Hayes, 2004). These combined therapies can help patients observe their thoughts from a more distanced and less reactive perspective, without getting caught up in them. Similarly, accepting thoughts and emotions without judging or trying to change them immediately helps individuals with neuroticism avoid internal struggles against their emotional experiences. Identifying levels of neuroticism and adjusting interventions accordingly—such as allowing them to choose between guided or silent meditation, or different types of practices like walking meditation or mindful yoga - can be beneficial. Introducing mindfulness practices gradually, starting with short sessions and increasing the duration as participants become more comfortable, can make MBIs more effective. Dialectical Behavior Therapy (DBT), the gold-standard treatment for Borderline Personality Disorder (BPD), integrates behavioral and mindfulness practices (focusing on the ability to manage emotions, tolerate distress, and improve interpersonal relationships), putting a particular focus on acceptance (Stiglmayr et al., 2014). Similarly, mindfulness-based cognitive therapy (MBCT) and mindfulness-integrated cognitive behavior therapy (MiCBT) are transdiagnostic approaches that focus on reducing avoidance and addressing interoceptive deficits and emotional reactivity. Accordingly, focusing on shared factors across psychological disorders and using transdiagnostic treatment protocols instead of multiple single disorder protocols can be a more resource-efficient approach for addressing comorbidity (Francis et al., 2024).

A systematic review examined the long-term effects and durability of mindfulness-based interventions (MBCT and MBSR) on mental health and well-being, using mediation analysis methods. Evidence was identified that mindfulness, rumination, and worry are significant mediators of the effects of MBIs on mental health outcomes, supporting the key theoretical premises underlying MBSR and MBCT. These findings suggest that cultivating mindfulness skills leads to understanding and acceptance of one’s own experience (Gu et al., 2015).

### 4.4 Limitations

Despite all the promising and positive findings, several limitations must be considered, and this review must be interpreted with these limitations in mind. Owing to the design of our review, we did not conduct a strict quality assessment of the studies, but there are some concerns that need to be discussed.

Currently, there is still debate about the conceptual and operational definitions of what “mindfulness” is and is not. MBI’s designed in one practice context may not be applicable in other contexts, because meaning is not transferred between settings. A scoping review identified four themes central to the concept of mindfulness, corresponding to the four domains of mindfulness research: mental health, behavioral change, cognitive neuroscience, and ethical mindfulness. However, operational definitions of mindfulness are not clearly articulated within these domains. Authors suggest greater attention should be given to developing operational definitions specific to each research domain, to avoid differing practices and definitions resulting in varied outcomes and benefits (Phan-Le et al., 2022).

Several methodological concerns were identified despite many of them not being new and having already been addressed (Davidson and Kaszniak, 2015). More than half of the included studies had an observational design, preventing causal inferences. Experimental studies often lacked follow-up assessments, had selection biases, or did not include active control groups. Moreover, most studies relied on self-reported measures, which introduced potential biases (e.g., social desirability and lack of objectivity). Along the same line, the heterogeneity of the instruments might complicate the task of comparing the results reported in the selected studies and once again reduce the generalizability of the findings. Several studies (Lee and Bowen, 2015; Iani et al., 2017; Heshmati and Pellerone, 2019; Kowalski and Schermer, 2019; Calvete et al., 2020) have also reported some concerns regarding the use of mindfulness instruments, as they were not certain that the instruments were assessing their desired outcome or were valid (Latzman and Masuda, 2013). As is the case with the FFMQ which gives a multifaceted view of mindfulness, compared with the MAAS that focus on a more general assessment of mindful attention, this fact may be contributing to inconsistent results (Hanley and Garland, 2017).

Sample characteristics also posed limitations. Participants were mostly young, educated, Caucasian females, limiting the generalizability of the findings. Future research should aim for more diverse samples and consider recruiting more male participants and individuals from various cultural backgrounds in mindfulness research (Bodenlos et al., 2017). It would be helpful to carry out interventions in areas traditionally dominated by men or male-oriented cultures, to recruit more male participants. An example is the study of the effects of an MBI on physiological and psychological criteria in a non-selective sample of police officers, where the results showed that participants with higher neuroticism and openness benefited more, and the effectiveness was greater for those who perceived a favorable social norm toward MBIs (Krick and Felfe, 2020). Furthermore, adapting MBIs for racial and ethnic minoritized communities could potentially make them more relevant and acceptable (Morales and Burnett-Zeigler, 2024). Low adherence to interventions among young people might be improved by incorporating technological supplements to the IMB, making the implementation more attractive and better suited for other study populations (Lucas-Thompson et al., 2020).

We believe it is crucial to focus mindfulness-based interventions on young adulthood (typically defined as mind-20 s), once brain maturation is largely complete. The prefrontal cortex, which plays a key role in emotional regulation and the modulation of personality traits like neuroticism, reaches full development during this period. Neuroticism predisposes individuals to depression by increasing the likelihood of ruminative responses to low mood (Barnhofer et al., 2011). Research indicates that mindfulness training can foster emotional regulation skills, reducing emotional reactivity and rumination, which are hallmark features of neuroticism (Kuehner et al., 2023). Introducing these interventions at this stage of life can be an effective preventive strategy to mitigate the manifestation of neuroticism throughout life.

Therefore, we recommend that mental health policies and professional practices incorporate mindfulness programs into educational and healthcare settings, specifically targeting young adults. This approach can help reduce the negative impact of neuroticism on mental health across the lifespan.

In the general adult population, facilitating the formation of practice groups where participants can share their experiences and support each other can be beneficial for improving adherence and overall well-being. Additionally, sampling methods were a limitation and likely a source of bias, as most studies used convenience samples. In the case of students, participation was compensated with academic credits. When sampling for experimental studies, there was a self-selection concern, as some studies recruited participants from populations that already had an interest in MBI interventions.

Future research should focus on developing high-quality, including randomized controlled trials, long-term follow-ups, recruiting larger and diverse samples, and exploring objective measures, to address these limitations.

## 5 Conclusion

Despite the limitations, these results can provide some insight and guide future research as well as future practice and policy-making decisions. A population-based study by Cuijpers et al. (2010) assessed the economic costs of neuroticism in a representative sample (*N* = 5,504) and found that the costs associated with neuroticism exceeded those of common mental disorders. Their results also suggest that there is a need to use interventions that target neuroticism as a cause of psychopathology, and here is where MBIs could serve as a useful intervention to aid in the development of abilities that can help people deal with the difficulties that neuroticism poses.

This evidence supports not only the relationship between neuroticism and mindfulness but also the effectiveness of MBIs in various mental and physical conditions, influencing core components shared with neuroticism. Targeting the development of behavioral and cognitive regulation skills may be particularly helpful in reducing neuroticism and thereby decreasing the risk of developing future affective disorders (Hanley and Garland, 2017).

One important question remains: Can neuroticism, as a personality trait, be changed through intervention? Cuijpers et al. (2010) proposed considering neuroticism as a fundamental component underlying mental disorders. Another question is to what extent mindfulness training can reduce neuroticism, even in adverse situations where past experiences and pain surface? Current literature trends emphasize a transdiagnostic approach to address the shared processes of mental disorders, improving the understanding of their heterogeneity and comorbidities (Dalgleish et al., 2020). A well-known transdiagnostic approach is the Unified Protocol (UP) (Barlow et al., 2017), an emotion-focused cognitive behavioral intervention that aims to target temperamental characteristics, especially neuroticism, by addressing mechanisms such as avoidance of emotional experience. It has shown promising results in the treatment of neuroticism (Sauer-Zavala et al., 2021), though further research is needed.

This review supports the relationship between neuroticism and mindfulness and their impact on mental and cognitive health. Despite the limitations, these findings provide insights for future research and practice. Emphasizing the need for more high-quality experimental studies is crucial, which will capture long-term follow-up studies, randomized controlled trials with larger and more diverse samples would be helpful. As well as the use of objective measures and integration with existing therapeutic frameworks could enhance our understanding and application of mindfulness interventions in addressing neuroticism-related health issues.

In conclusion, the negative correlation between neuroticism and its core components (e.g., negative mood, anxiety, and depression) with mindfulness and their impact on mental health and cognitive responses are evident. Future research should explore the clinical implications of these findings in high-quality experimental studies to further validate and expand upon these promising results.

## Data Availability

The original contributions presented in the study are included in the article/Supplementary material, further inquiries can be directed to the corresponding author.
